# A case of chronic actinic dermatitis that responded completely to treatment with oral colostrum‐macrophage‐activating factor (colostrum‐MAF)

**DOI:** 10.1111/phpp.12469

**Published:** 2019-04-29

**Authors:** Masamitsu Ichihashi, Yoshitaka Nakamura, Masahiko Muto, Takahito Nishikata, Tosio Inui, Yoshihiro Uto

**Affiliations:** ^1^ Arts Ginza Clinic Tokyo Japan; ^2^ Department of Dermatology Yamaguchi University Ube Japan; ^3^ Department of Dermatology Ube‐Kosan Central Hospital Ube Japan; ^4^ Graduate of Frontiers of Innovative Research in Science and Technology (FIRST) Konan University Kobe Japan; ^5^ Saisei Mirai Clinic Kobe Kobe Japan; ^6^ Graduate School of Technology, Industrial and Social Sciences Tokushima University Tokushima Japan

A 41‐year‐old Japanese male patient with a half‐year history of pruritic severe erythema on his face, neck, trunk, and upper extremities, specifically on his sun‐exposed areas, was diagnosed with chronic actinic dermatitis (CAD) at the Department of Dermatology at Yamaguchi University Hospital and was referred to the Saisei Mirai Clinic in Kobe, Japan, in September 2015. He was hypersensitive to UVA at a dose of 1.7 J/cm^2^ (tested with a TOREX/FL20S‐BL/DMR, Toshiba emitting light ranging from 315 to 410 nm, peaking at 360 nm; normal minimal erythema dose = >10‐15 J/cm^2^), but had a normal response to UVB (minimal erythema dose: 114 mJ/cm^2^, tested with a Philips TL20W/12RS emitting light from 270 to 360 nm, peaking at 310 nm; normal minimal erythema dose = 60‐100 mJ/cm^2^). The patient did not respond to a low dose (5 mg/d) of systemic corticosteroid hormone or to various topical treatments with steroid hormones and a calcineurin inhibitor, tacrolimus, prescribed at other hospitals, for more than half a year. The patient had not taken any medication capable of inducing photosensitivity before the onset of CAD.

Physical examination at his first visit to the Saisei Mirai Clinic in September 2015 revealed severe erythema with partly exudative, crusted lesions, and scratch marks, similar to the characteristics of atopic dermatitis, on his face. Further, he had Hertoghe's sign, often observed in severe atopic dermatitis, and diffuse pigmentation on his face and erythema and pigmentation on his neck but was free from eruptions on his lower extremities. He was found to suffer from cataracts in both eyes.

In September 2015, the patient started to take two capsules daily of colostrum‐macrophage‐activating factor (colostrum‐MAF), which is a health food containing degalactosylated/desialylated bovine colostrum frequently used successfully by patients with other indications such as atopic dermatitis and autism. He was also treated topically with weak and strong steroid hormone ointments on his face and neck, and on his trunk and extremities, respectively. Each capsule contained 1 mg colostrum‐MAF and other non‐identified components. After 2 weeks of treatment, he stopped the topical treatment since the itchy erythema seen on most of his body except his lower extremities had improved dramatically, although diffuse pigmentation remained (Figure 1A). The patient continued to take oral colostrum‐MAF every day for nearly 10 months. After the cessation of the oral treatment with colostrum‐MAF, the patient was free from erythema for 4 months and used only sunscreen for his daily care, and finally, he began to work outdoors in the daytime.

**Figure 1:**
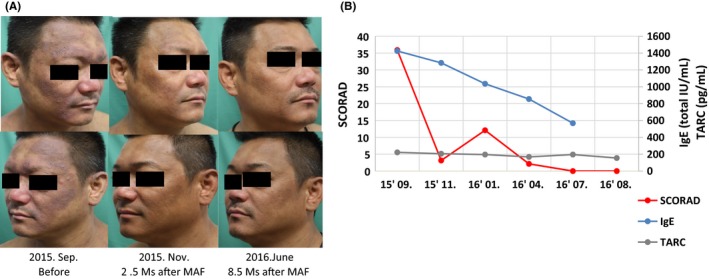
A, Clinical photographs of the 41‐year‐old male patient with CAD, taken before colostrum‐MAF treatment (September 2015), after 2.5 mos of treatment (November 2015), and after 8.5 mos of colostrum treatment (June 2016). B, On examination, his SCORAD score decreased over the first month of colostrum‐MAF treatment in parallel with his clinical improvement. Further, his serum IgE level also decreased significantly

Blood cell counts, and liver and kidney functions were all within normal limits. Serum IgE and TARC (thymus and activation‐regulated chemokine) were 1425 IU/mL and 222 pg/mL at his first visit (September 2015) and were 590 IU/mL and 206 pg/mL at his visit on July 2016 to the Clinic, respectively. Eosinophils were in the normal range. His SCORAD index improved from 36.0 at his first observation to 0.4 at his last visit (Figure 1B).

Colostrum is rich in immunoglobulins IgA, IgG, and IgM and is expected to modulate immunity, since IgA has an O‐linked sugar chain similar to that in group‐specific component (Gc) protein, a precursor of Gc protein‐derived macrophage‐activating factor (Gc‐MAF), which is produced from colostrum Gc protein by cleaving sialic acid and β‐galactoside.^1^, ^2^ Further, we have found that colostrum‐MAF has a suppressive effect on the LPS/IFN‐γ‐induced expression of TNF‐α (Figure 2A) and increased the intensity of CD206 (a marker of M2 macrophages) similar to that induced by IL‐4/IL‐13 stimulation (Figure 2B). Those results suggest that colostrum‐MAF may play a role in immune modulation by activating type 2 macrophages with regulatory functions.^3^

**Figure 2:**
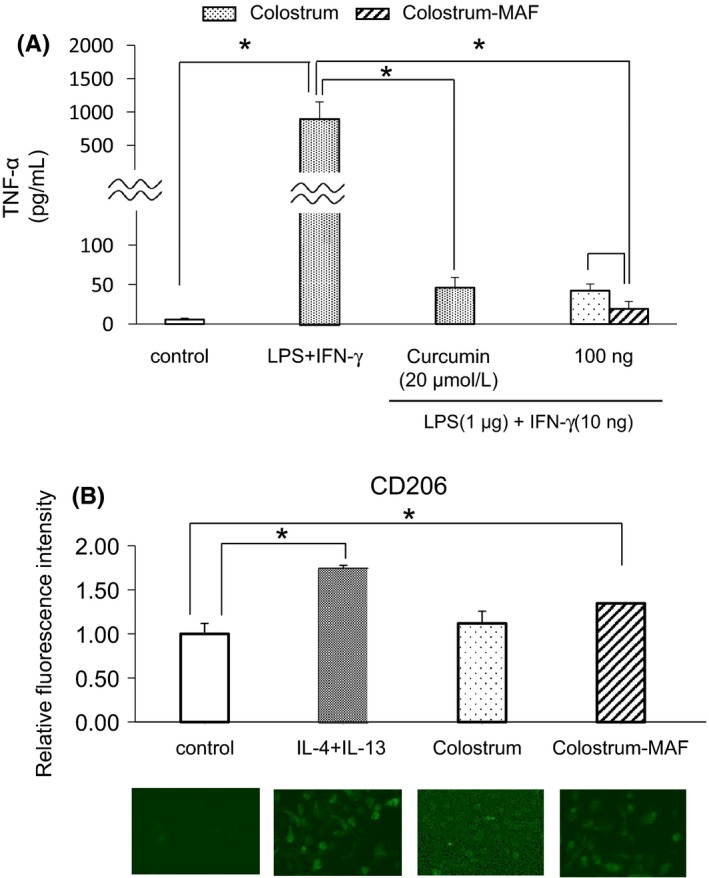
A, Suppressive effect of colostrum‐MAF against TNF‐α production induced by LPS and IFN‐γ. Mouse peritoneal macrophages were cultured in 24‐well plates at a density of 5 × 10^5^ cells/well in serum‐free RPMI 1640 for 18 h. The cultured cells were washed two times with serum‐free RPMI and were then treated with LPS (1 μg) + IFN‐γ (10 ng) with or without colostrum (100 ng) or colostrum‐MAF (100 ng) for 24 h. The supernatants were then collected and assayed with an ELISA kit for Mouse TNF‐α (ELISAReady‐SEF‐Go). The combination of LPS (1 μg) and IFN‐γ (10 ng) significantly induced TNF‐α production by mouse peritoneal macrophages. In contrast, the addition of colostrum (100 ng) or colostrum‐MAF (100 ng) to LPS + IFN‐γ significantly decreased the production of TNF‐α, just like curcumin (20 μM) that was used as a positive control. Data are expressed as means and standard deviations from three independent experiments. The statistical significance was determined by Student's *t* test. *P < 0.05 B. Effect of colostrum‐MAF on the polarization of M2 macrophages. Mouse peritoneal macrophages were treated with IL‐4 (30 ng) + IL‐13 (30 ng), colostrum (10 ng), or colostrum‐MAF (10 ng) for 24 h. After fixation with methanol for 10 min, the cells were dried and incubated overnight with 1 mL 1% BSA at 4°C. After washing with PBS, immunocytochemical staining was performed for macrophage mannose receptor (CD206) on permeabilized cells to visualize cell surfaces. Treatment with IL‐4 and IL‐13 (each 20 ng) was used as a positive control to induce M2 macrophages. Colostrum‐MAF (100 ng) significantly induced M2 macrophages but in contrast, colostrum alone (100 ng) did not induce M2 macrophages. All experiments were performed in triplicate, and data are reported relative to the fluorescence intensity of the control. Each error bar represents the standard deviation. *P < 0.05

Chronic actinic dermatitis is a rather rare photosensitive disease commonly affecting elderly men^4^ and often is difficult to differentially diagnose from photoaggravated dermatitis, although CAD can arise in young people with pre‐existing dermatoses, such as allergic contact dermatitis, atopic dermatitis, and HIV infection.^5^ An alternative diagnosis in this patient was severe photoaggravated dermatitis, especially as the patient's sensitivity was to UVA rather than UVB.

The exact pathological mechanism of CAD still remains to be clarified. It had been regarded as a contact dermatitis‐like reaction,^6^ but Ko et al^7^ recently proposed that CAD may be caused by a Th1/Th2 dysbalance, based on the positive relationship between clinical severity and total IgE level and eosinophilia in the peripheral blood of patients with CAD.

For the correct diagnosis of CAD, photosensitivity tests using an artificial light source from UVB to visible light are essential, and patch tests using European Standards Allergen Series plus sunscreens, corticosteroids cosmetic series, and photo‐patch tests are also recommended.^6^, ^7^ In this case, a patch test was not performed, since the patient did not agree to that test.

For clinical management, the avoidance of active wavebands is basically the most important. To manage acute eczematous dermatitis, topical use of corticosteroid‐ or calmodulin inhibitor‐containing ointments is commonly recommended, but these topical treatments are not so effective in most patients with CAD. The present 41‐year‐old male patient was photosensitive to UVA and was refractory to topical treatment with the strongest class corticosteroids for more than 3 months and to oral intake of small amounts of predonisolone (5 mg/d) for approximately 2 months. Severe exudative erythema with scratch marks on his face responded quite well to oral uptake of two capsules of bovine colostrum‐MAF. The exact amount of colostrum‐MAF contained in each capsule is calculated to be around 1 μg based on the conversion rate of human Gc‐MAF (group‐specific‐macrophage‐activating factor) from Gc protein by enzymatic cleavage. Severe erythema significantly subsided on his second visit after initiation of colostrum‐MAF treatment with supportive short‐term (5‐7 days) application of steroid hormone or tacrolimus ointment and oral intake of anti‐allergic agent for 2 weeks. After 9 months of treatment, the UVA hypersensitivity disappeared.

Clinical and laboratory characteristics strongly suggest that the main cause of CAD may be immunological, although the detailed mechanism still remains to be clarified. Patients with CAD show a Th‐2 polarization with the co‐existence of tissue eosinophilia and disease severity,^8^ and further, Ko et al^7^ recently suggested that a Th1/Th2 dysbalance caused by suppressor T cells may play a role in CAD occurrence.

In the present study, we found that colostrum‐MAF increased the number of and activated M2 macrophages, but not M1 macrophages, and significantly suppressed LPS‐induced inflammatory cytokines activation in an in vitro study of mouse intra‐peritoneal macrophages. These findings suggested that colostrum‐MAF may modulate immune dysfunction in allergic skin diseases, such as atopic dermatitis, and in photosensitive diseases including CAD and polymorphous light eruptions. Surprisingly, the present patient with CAD responded quite well to the oral intake of bovine colostrum‐MAF even after only 2 weeks, and severe and erythema refractory to conventional therapies almost disappeared after 2 months of treatment. Based on our in vitro study and recent reports by others, we speculate that colostrum‐MAF may modulate M1/M2 macrophage polarization, leading to the subsidence of inflammatory reactions in the skin. To recommend the general use of colostrum‐MAF on inflammatory skin diseases will require further clinical studies on a number of cases to confirm the efficacy and safety with optimal dose for each disease in the future.

## CONFLICT OF INTEREST

The authors, except Dr. Inui T and Prof. Uto Y, have no conflict of interest to declare. Dr. Inui T is President of Saisei Mirai Clinic where colostrum‐MAF is produced. Prof. Uto Y is supported by Dr. Inui for his study.
